# Causes of Pre-hospital Delay in Acute Stroke in Punjab

**DOI:** 10.7759/cureus.39180

**Published:** 2023-05-18

**Authors:** Amit Kumar Gupta, Kamaldeep Kaur, Lovleen Bhatia, Rupinderjeet Kaur, Ajay Bhaskar, Gurpreet Singh

**Affiliations:** 1 Department of Cardiology, Dayanand Medical College & Hospital, Ludhiana, Ludhiana, IND; 2 Department of Medicine, Government Medical College and Rajindra Hospital Patiala, Patiala, IND; 3 Department of Medicine, Government Medical College, Patiala, Patiala, IND

**Keywords:** development of symptoms, start of treatment, thrombolytic therapy, stroke, pre-hospital delay

## Abstract

Background

Pre-hospital delay, which refers to the time delay between the development of symptoms in the patient and the start of treatment, is one of the major factors impacting the treatment of stroke. This study aimed to identify patient characteristics and factors causing a pre-hospital delay in acute stroke (both ischemic and hemorrhagic) cases.

Methodology

This prospective follow-up study included 100 patients who presented with clinical features of acute stroke within 48 hours of symptom onset. A pre-designed questionnaire was administered within 72 hours of hospital admission to every patient.

Results

The mean time to hospital presentation was 7.73 hours. Only 2% of patients were thrombolysed. Age group, gender, education status, occupation, and socioeconomic status were not significantly (p > 0.05) associated with the mean symptom onset time to hospital arrival. Rural area (p < 0.001), nuclear family (p = 0.004), distance from the tertiary care center (p < 0.001), being alone at the time of symptom onset (p < 0.001), lack of knowledge about symptoms of stroke in patient/attendant (p < 0.001), and mode of transport were the factors that emerged as significant predictors of pre-hospital delay on univariate analysis. Living in a nuclear family, distance from the tertiary care center, and mode of transport were the factors that emerged as independent predictors of pre-hospital delay on multiple linear regression analysis.

Conclusions

In this study, factors associated with delayed hospital presentation including living in a nuclear family, distance from the tertiary care center, and use of public transport to reach the hospital emerged as independent predictors of pre-hospital delay.

## Introduction

Stroke is “a clinical syndrome characterized by rapidly developing clinical signs of focal (or global) disturbance of cerebral function, lasting more than 24 hours or leading to death, with no apparent cause other than that of vascular origin” [1]. Stroke is a significant global health issue that can have severe and long-lasting effects on individuals and communities. Worldwide, accounting for 11% of total deaths, stroke ranks second in the causes of death. Apart from the high mortality rate, stroke causes major impacts on the affected individuals due to long-term disability [2]. The burden of stroke differs across regions, with a major burden in developing and underdeveloped countries. However, stroke remains a major health issue, even in developed nations, and the disease burden has been increasing over the years because of the raising burden of factors such as obesity, diabetes, hypertension, and an aging population. The effect of stroke on global health extends beyond individual health outcomes, with significant economic and social costs. Stroke can lead to long-term disability, which can result in reduced quality of life, decreased productivity, and increased healthcare costs. Particularly in developing and underdeveloped countries, the economic impact of stroke is significant as the cost of stroke care can place a substantial burden on already limited healthcare resources [3].

Stroke is a significant public health issue in India. According to a study published in the Lancet in 2016, stroke ranks second in the cause of death in India, with 8.4% of total deaths [4]. According to the current international burden of disease statistics on stroke, in 2013, there were nearly 25.7 million stroke survivors, 6.5 million deaths, 113 million disability-adjusted life years because of stroke, and 10.3 million newly diagnosed stroke patients [5]. The high problem of stroke is partially due to poor public awareness of stroke hazard factors and the warning symptoms. This is maintained by the indication that amplified knowledge of stroke risk factors leads to enhanced compliance with stroke inhibition practices, although lack of recognition of warning symptoms of stroke is a significant contributory aspect of delay in healthcare center reporting of stroke [6].

Pre-hospital delay, which refers to the time delay between the development of symptoms in the patient and the start of treatment, is one of the major factors impacting the treatment of stroke. The faster the patient receives medical attention, the chances of a positive outcome increase. Therefore, reducing pre-hospital delay is a crucial factor for better outcomes. Pre-hospital delay is a significant issue in stroke management [7]. Very few studies evaluating the causes of pre-hospital delay have been reported in India, where the problem is increased by huge rural populations, low health literacy, and other factors. This study aimed to identify such factors in the Indian context by studying the relationship of pre-hospital delay with patient characteristics and situational factors in acute stroke. This may help us in developing strategies to reduce pre-hospital delay and improve the health services delivered to stroke patients.

## Materials and methods

The study was conducted over one year from November 2018 to October 2019 at the Department of Medicine, Government Medical College and Rajindra Hospital Patiala after obtaining approval (ethical committee approval number: BFUHS/2k17p-TH/14736). This prospective follow-up study was conducted to identify the related factors causing a pre-hospital delay in acute stroke. A total of 100 consecutive acute stroke cases who presented to the hospital with a confirmed diagnosis of acute stroke and who fulfilled the inclusion criteria were included as study participants. Cases where reliable history could not be obtained from the patient or the attendant and patients presenting after 48 hours of symptom onset were not included in the study. Stroke onset time was defined as the time when the first symptom developed in the patient. The hospital presentation time was defined as the time when the stroke patient was assessed by the clinician at the hospital. The pre-hospital delay was calculated based on the difference between these two. A written and informed consent form was obtained from each patient (if possible) or attendant. A pre-designed questionnaire was administered within 72 hours of hospital admission to every patient. In the case of dysphasic patients or those with altered consciousness, the questionnaire was administered to the primary caregiver or attendant.

Statistical analysis

SPSS version 22 (IBM Corp., Armonk, NY, USA) was used to perform data analysis. The dependent variable (time to hospital presentation) was normally distributed. Univariate analysis was done between the dependent and independent variables (living in a nuclear family, distance from the tertiary care center, and the use of public transport ). The t-test was used for factors with two groups, and analysis of variance was used for factors with more than two groups. Multiple linear regression analysis was done taking mean time to hospital presentation as the dependent variable, and other significant variables found on univariate analysis as predictors (independent variables), to determine the independent effect of significant variables.

## Results

A total of 100 acute stroke cases selected through consecutive sampling method were included. Table 1 describes the study population. The majority of the acute stroke patients (50%) were between 66 and 80 years of age, and most of them (64%) were males. Overall, 28% of patients were illiterate, while 49% of patients were educated only up to primary school. Most study participants belonged to the lower middle class (41%) and lived in rural areas (76%).

**Table 1:** Characteristics of the study population.

Variables	N = 100
Age (in years)	
<50	7
50–65	31
66–80	50
>80	12
Gender (%)	
Male	63
Female	36
Education status	
Illiterate	28
Completed primary school	49
Completed secondary school and above	23
Socioeconomic group	
I	0
II	15
III	41
IV	24
V	20
Place of residence	
Rural	76
Urban	24
Time from symptoms onset to hospital presentation (hours)	
≤3	6
3–4.5	7
4.5–6	19
6–12	56
12–48	12

Regarding symptom onset to hospital presentation time, only 6% of the cases reached within three hours to the hospital. The majority of them (56%) arrived between six and 12 hours, and 12% of patients reported after 12-48 hours of symptom onset.

Table 2 shows the analysis of the variables with mean hospital presentation time. The average time to hospital presentation for males was 7.71 hours (95% confidence interval (CI) = 6.94-8.51 hours), while for female patients, it was 7.75 hours (95% CI = 6.66-8.83 hours). The mean time to hospital presentation in employed patients was 7.6923 hours (95% CI = 6.2930-9.0916 hours), whereas in unemployed/retired patients, this time was 7.7432 hours (95% CI = 7.0405-8.4595 hours). No statistically significant difference was found between variables, such as gender, age group, socioeconomic status, and education level, and mean time to hospital admission.

**Table 2:** Analysis of the variables with the mean hospital presentation time. *: Denotes significant (p < 0.05). NIHSS: National Institutes of Health Stroke Scale; TIA: transient ischemic attack

Variable	N (100)	Time to hospital presentation hours (Mean ± SD)	P-value
Sex			
Male	64	7.71 ± 3.14	0.962
Female	36	7.75 ± 3.21	
Age group			
<50	7	7.42 ± 2.37	0.74
50–65	31	7.40 ± 3.83	
66–80	50	8.09 ± 2.95	
>80	12	7.29 ± 2.54	
Employment status			
Employed	26	7.69 ± 3.46	0.944
Unemployed/Retired	74	7.74 ± 3.06	
Education status			
Illiterate	28	8.69 ± 2.49	0.147
Completed primary school	49	7.24 ± 3.52	
Completed secondary school and above	23	7.58 ± 2.87	
Socioeconomic classification			
II	15	6.50 ± 4.01	0.057
III	41	7.19 ± 2.79	
IV	24	8.43 ± 1.74	
V	20	8.90 ± 3.98	
Place of residence			
Rural	76	8.49 ± 2.48	<0.001*
Urban	24	5.33 ± 2.78	
Gender of the attendant			
Male	92	7.62 ± 3.21	0.239
Female	8	9.00 ± 2.01	
Education status of the attendant			
Up to primary school	28	7.50 ± 2.56	0.53
Up to secondary school	64	7.91 ± 3.42	
Graduate and above	8	6.25 ± 1.65	
Day of presentation			
Weekend	12	8.58 ± 3.41	0.32
Weekday	88	7.61 ± 3.12	
Time of onset of symptoms			
Day (06:00 to 18:00)	46	7.27 ± 2.92	0.176
Night (18:00 to 06:00)	54	8.12 ± 3.31	
Setting where symptoms occurred			
Home	86	7.66 ± 3.07	0.622
Workplace	6	7.33 ± 3.84	
Outside	8	8.75 ± 3.75	
Living arrangement			
Joint family	84	7.34 ± 2.96	0.004
Nuclear family	16	9.78 ± 3.43	
Distance from the tertiary care center (km)			
<30	26	4.17 ± 1.39	<0.001*
30–60	57	7.99 ± 1.86	
>60	17	12.32 ± 1.75	
Witness to symptom onset			
Alone	55	9.02 ± 3.02	<0.001*
Family member	40	6.23 ± 2.67	
Co-worker	5	5.50 ± 0.93	
Initial appraisal of symptoms by the patient			
Serious	36	5.06 ± 1.45	<0.001*
Not serious	40	9.90 ± 2.37	
Could not be assessed	24	8.12 ± 3.23	
Previous knowledge about symptoms of stroke in the patient			
Yes	28	4.94 ± 1.36	<0.001*
No	48	9.16 ± 2.81	
Could not be assessed	24	8.12 ± 3.25	
Directly reaching the tertiary center			
Yes	22	4.20 ± 1.60	<0.001*
No	78	8.73 ± 2.74	
NIHSS score			
≤5	10	8.70 ± 5.46	<0.001*
6–15	79	7.96 ± 2.96	
>15	11	5.22 ± 1.97	
Type of stroke			
Ischemic	87	8.18 ± 3.02	<0.001*
Hemorrhagic	11	5.22 ± 1.97	
TIA	2	1.75 ± 0.35	
Mode of transport			
Private vehicle	12	2.95 ± 0.83	<0.001*
Ambulance	44	6.42 ± 1.66	
Public transport	44	10.35 ± 2.19	

In this study, patients living at a longer distance from the tertiary care center had a longer pre-hospital delay. Thus, distance from the tertiary care center was found to be significantly associated with time to hospital presentation on univariate analysis (p < 0.001). Moreover, this factor emerged as an independent factor affecting time to hospital presentation on multiple linear regression analysis (p = 0.000). This is due to the long tapping area of the study hospital, which is a premier tertiary care hospital. However, witnessing symptom onset did not emerge as an independent factor affecting time to hospital presentation on multiple linear regression analysis.

In this study, 36 out of 100 patients appraised the symptoms as serious while 40 patients appraised the symptoms as non-serious. In 24 patients, we could not enquire about the initial appraisal of symptoms due to poor general condition or altered mentation of the patient.

Furthermore, 28 of 100 patients had previous knowledge about the symptomatology of stroke. The mean time to hospital presentation in this group was 4.9464 hours (95% CI = 4.4152-5.4776 hours). While 48 patients had no such knowledge, and the mean time in them was 9.1667 hours (95% CI = 8.3507-9.9827 hours). In 24 patients, this could not be assessed due to poor general condition or altered mentation, and the mean time in them was 8.1250 hours (95% CI = 6.7521-9.4979 hours). The difference between the groups was statistically significant (p < 0.001) on univariate analysis, but previous knowledge about the symptomatology of stroke did not emerge as an independent factor affecting time to hospital presentation on multiple linear regression analysis.

However, a statistically significant difference (p < 0.001) was noted between variables such as place of residence, living arrangement, distance from the tertiary care center, witnessing symptom onset, initial appraisal of symptoms by patient, previous knowledge about symptoms of stroke in patient, National Institutes of Health Stroke Scale (NIHSS) score, type of stroke, and mode of transport and mean time to hospital admission. The difference between the groups was statistically significant (p < 0.001) on univariate analysis, but the NIHSS score did not emerge as an independent factor affecting time to hospital presentation on multiple linear regression analysis.

The mean hospital presentation time in those living in a joint family was 7.3452 hours (95% CI = 6.7023-7.9882 hours), and for those living in a nuclear family, the average hospital presentation time was 9.7812 hours (95% CI = 7.9535-11.6090 hours). The average hospital presentation time in individuals with prior knowledge about stroke onset was 4.9464 hours (95% CI = 4.4152-5.4776 hours). While 48 patients had no such knowledge, and the mean time in them was 9.1667 hours (95% CI = 8.3507-9.9827 hours).

In this study, 12 patient used their own or an attendant’s private vehicle, 44 used an ambulance, and the remaining 44 used public transport to reach the hospital. The mean time to hospital presentation in each group was 2.9583 hours (95% CI = 2.4257-3.4909 hours), 6.4205 hours (95% CI = 5.9147-6.9262 hours), and 10.3523 hours (95% CI = 9.6856-11.0190 hours), respectively. Among the study participants, 87 patients were diagnosed with an ischemic stroke, 11 patients were diagnosed with a hemorrhagic stroke, and two patients were diagnosed with a transient ischemic attack. The mean time to hospital presentation in each group was 8.1897 hours (95% CI = 7.5451-8.8342 hours), 5.2273 hours (95% CI = 3.8975-6.5571 hours), and 1.7500 hours (95% CI = 1.4266-4.9266 hours), respectively.

Table 3 describes the analysis of stroke risk factors with mean hospital presentation time. No statistically significant (p > 0.05) difference was noted in the mean hospital presentation time and risk factors. In total, 33 patients consumed indigenous opioid-like products (bhukki/affem). The mean time to hospital presentation in these patients was 7.9848 hours (95% CI 6.8389-9.1308 hours), while in the remaining 67, it was 7.6119 hours (95% CI = 6.84808.3759 hours).

**Table 3:** Analysis of stroke risk factors with mean hospital presentation time. *: Denotes significant (p < 0.05).

Risk factor	Number of patients		Mean time to hospital presentation (hours)	Standard deviation	P-value
Diabetes mellitus	Yes	19	7.31	3.85	0.522
	No	81	7.83	2.98	
Hypertension	Yes	53	7.94	3.18	0.486
	No	47	7.50	3.13	
Coronary artery disease	Yes	17	7.26	2.80	0.503
	No	83	7.83	3.22	
Prior history of stroke	Yes	8	5.50	0.75	<0.001*
	No	92	7.92	3.20	
Smoking	Yes	23	7.02	3.24	0.218
	No	77	7.94	3.11	
Opioid abuse	Yes	33	7.98	3.23	0.581
	No	67	7.61	3.13	

In this study, the cut-off time for thrombolysis was kept at 4.5 hours. However, we did not evaluate In-hospital delay, which also needs to be taken into account while considering thrombolysis. This delay, measured in terms of emergency department arrival to recombinant tissue plasminogen activator administration, has been estimated to vary from 0.8 hours [8] to 1.5 hours [9]. Taking pre-hospital and in-hospital delay into consideration, only two out of the 100 patients were thrombolysed because most patients arrived out of the window period for thrombolysis.

## Discussion

Despite recent advances in stroke management, a major percentage of acute stroke cases do not seek immediate medical attention. Even in high-income countries, a lacuna exists in the awareness among stroke patients regarding warning signs and symptoms [10]. The therapeutic window for thrombolytic treatment is <4 1/2 hours and favorable outcomes can be achieved if the treatment is administrated within 90 minutes [11]. Despite these, only a few cases of acute stroke undergo thrombolytic treatment due to excessive pre-hospital delay.

In this study, the cut-off time for thrombolysis from symptom onset was taken as 4.5 hours. In our study, only 2% of the patients received were thrombolysed because the time presentation for others was outside the window period for thrombolysis. The percentage of acute stroke cases who receive thrombolytic therapy is relatively low, even in developed countries. A study conducted in the United States reported that only 2-7% of ischemic stroke patients receive thrombolytic therapy and that the use of such therapy is associated with improved outcomes [12]. However, a recent report showed that the proportion of eligible patients who received therapy increased from 4% in 2010 to 20% in 2016 in the United Kingdom [13]. The mean time to hospital presentation in our study was 7.73 hours.

Lees et al. noted that the average time to administer intravenous alteplase in stroke patients after symptom onset enrolled in four clinical trials was 3.4 hours [14]. However, the study noted that the time to treatment varied widely among patients, ranging from less than one hour to more than 12 hours [14]. A recent study conducted in 2021 reported a pre-hospital delay of 283 minutes (just under five hours) [15]. The study noted that increased pre-hospital delay was associated with old age, female sex, non-lacunar infarction, and lack of knowledge of stroke symptoms [15]. Another recent study also reported that the average time to hospital arrival in acute stroke cases who received thrombolytic treatment was 4.4 hours compared to 13.2 hours in patients who had not received thrombolytic treatment. The study noted that longer admission delay was associated with worse in-hospital outcomes in both groups of patients [16].

Stevens et al. reported that the longer time to arrival was associated with increasing age, female gender, and lower income [17]. However, in our study, age group, gender, education status, occupation, and socioeconomic status were not significantly associated with pre-hospital delay.

In our study, living in a rural area, living alone, the distance from the tertiary care center, being alone at the time of symptom onset, inadequate knowledge about stroke symptoms in the patient/attendant, and mode of transport emerged as significant predictors of pre-hospital delay on univariate analysis. Rural-to-urban differences in the timing of presentation to hospitals have been reported in studies conducted in India [18,19]. Ashraf et al. found no significant urban-to-rural differences. However, a study reported that rural patients were more likely to use traditional medicine or seek care from non-allopathic practitioners before presenting to the hospital [20].

For patients living in a joint family at the time of symptom onset, the family members may help in symptom recognition and initial appraisal. They also help in arranging transportation. In this study, patients from a nuclear family had a longer pre-hospital delay. However, this result was in contradiction with previous Indian studies reported by Srivastava et al. [18] and Pandian et al. [21]. Unlike in Western countries, where emergency medical services are readily available, negating the importance of distance is not feasible in our country. In this study, patients having a longer distance from the tertiary care center had a longer pre-hospital delay. Many previous studies reported similar findings [19,21-23]. Inadequate knowledge regarding stroke symptoms and early treatment was found to be a significant barrier to timely recognition and response to stroke symptoms. A qualitative review of 26 studies on knowledge and awareness about the symptoms of stroke found that inadequate knowledge regarding symptoms and lack of knowledge were associated with longer pre-hospital delay [24]. Similar observations have been reported by other studies [25,26]. Overall, all previous studies suggest that the mean time of presentation to hospitals after symptom onset in stroke patients varies widely depending on various factors.

Study limitations

We acknowledge the limitations of the present study. First, it is a single-center study using a small sample size. However, it provides a useful understanding of issues surrounding pre-hospital delay. More multicentric studies with a larger number of patients are needed to confirm our findings.

## Conclusions

Intravenous thrombolytic therapy has proven to be a safe and effective treatment in patients with acute ischemic stroke. However, the therapeutic window is narrow, extending up to 4.5 hours. Only a minority of patients get admitted to the hospital in this narrow therapeutic window because of delayed admission, which deprives them of this life-saving therapy. Pre-hospital delay is a major component of this delay. Very few studies from India have evaluated pre-hospital delay where local sociodemographic, clinical, cognitive, and emotional factors interplay to alter the time to hospital presentation. The mean time to hospital presentation in this study was 7.73 hours. Living in a nuclear family, distance from the tertiary care center, and the use of public transport as the transport medium to reach the hospital emerged as independent predictors of pre-hospital delay.
